# Soft Tissue Ewing Sarcoma in a Child: A Rare Localization

**DOI:** 10.7759/cureus.103374

**Published:** 2026-02-10

**Authors:** Larbi Benradi, Hanane Salhi, Mohamed Belahcen

**Affiliations:** 1 Pediatric Surgery, Mohammed VI University Hospital of Oujda, Oujda, MAR; 2 Pediatric Surgery, CHU Mohammed VI, Oujda, MAR; 3 Faculty of Medicine, Mohammed 1st University of Oujda, Oujda, MAR

**Keywords:** child, ewing sarcoma, multidisciplinary, soft tissue, surgery

## Abstract

Ewing sarcoma (ES) is a primitive neuroectodermal tumor usually located in the bones. The soft tissue as a primary localisation of ES is extremely rare. A few cases have been reported in the literature; the most affected areas are the head, trunk, neck, upper and lower limbs, or even multiple lesions. It is most often a painful and mobile subcutaneous swelling with a soft consistency; metastasis is very rare. Differential diagnosis is made with other small round cell neoplasms. Therapy for ES includes chemotherapy, radiation therapy, and surgery. In this paper, we discuss the findings of a soft tissue ewing sarcoma (STES) located in the right arm in a 14-year-old boy successfully treated in our department of pediatric surgery. The follow-up did not show any sign of recurrence after 24 months.

## Introduction

Ewing sarcoma (ES) is an uncommon and highly aggressive malignant bone tumor that predominantly occurs in children and young adults, typically in the second and third decades of life, and shows no gender preference. It is the second most common bone tumor in order of frequency after osteosarcoma [1,2]. ES most commonly arises from the diaphysis of long bones [3]. ES rarely arises from soft tissues, with only a handful of cases documented across different parts of the body, while the upper and lower limbs remain the most frequent sites [4].

The clinical presentation of soft tissue Ewing sarcoma (STES) is highly variable and depends on the location of the mass. Biopsy with pathology and immunohystochemistry are crucial for the diagnosis of STES. Most of extraskeletal ES (EES) show CD99 positivity, but this marker is not entirely specific; therefore, additional markers, such as NKX2.2, are employed to differentiate ES from other small round-cell tumors [5]. The fusion of the ES breakpoint region 1 (EWSR1) and friend leukemia virus integration 1 (FLI1) is present in 90% of ES cases [3].

Treatment consists of radical surgical excision of the mass after or even without chemotherapy, followed by radiotherapy of the tumor bed and/or postoperative chemotherapy. However, there is no standard treatment yet [6].

We present the findings of a soft tissue ES located in the right arm of a 14-year-old boy managed at our academic center. After 24 months of clinical and radiological follow-up, no signs of recurrence were observed.

## Case presentation

**Figure 1:**
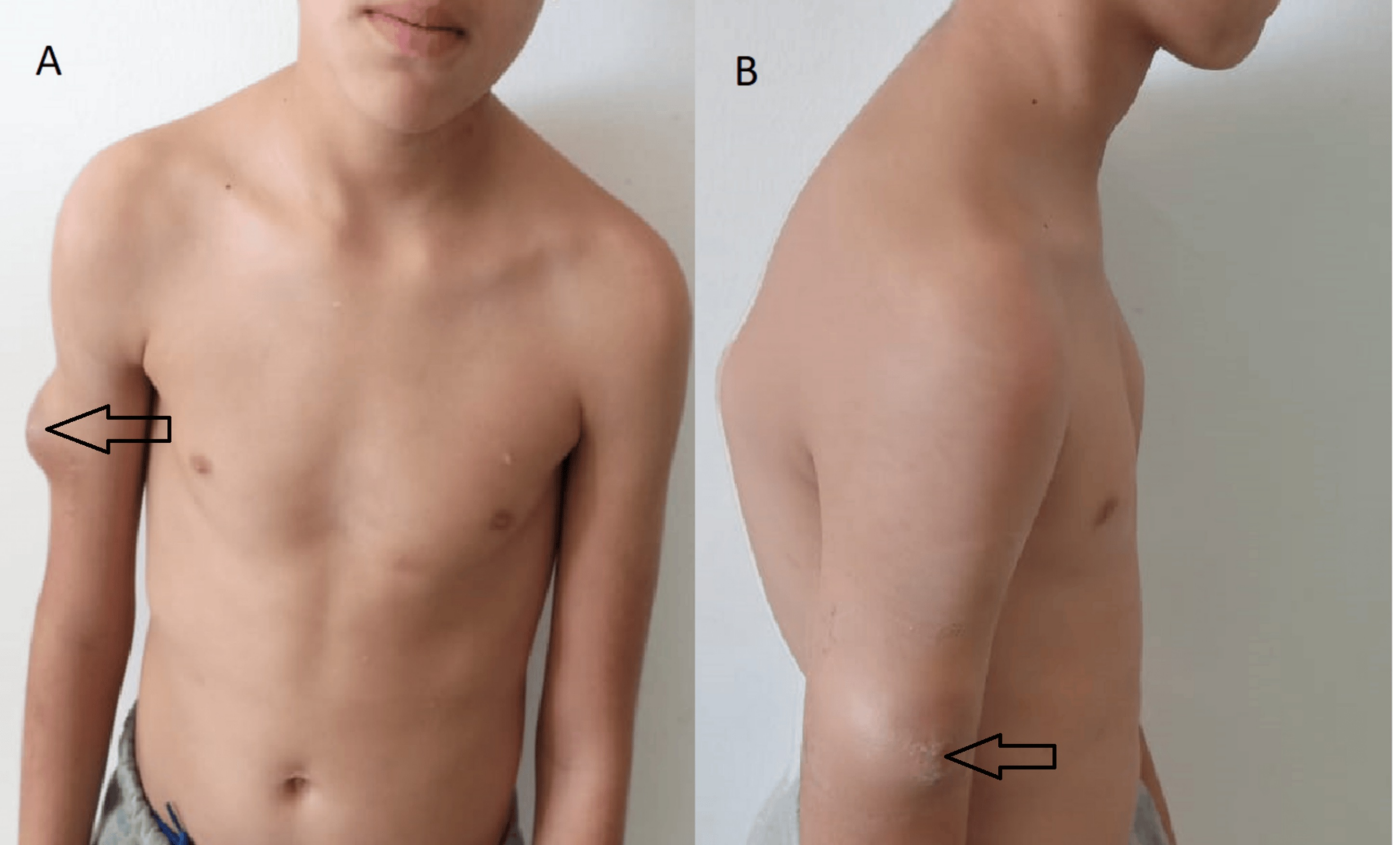
A, B clinical images showing lateral superficial subcutaneous right arm swelling ( black arrows) measuring 7 x 5 cm.

A 14-year-old boy with no significant past medical history was referred by his family physician to our Department of Pediatric Surgery for a painful swelling of the right arm that had appeared six months earlier and had been gradually increasing in size. The patient had no relevant family or drug history, no prior surgeries, and no known genetic predisposition to disease. Clinical examination revealed an afebrile child in good general condition. There was no evidence of weight loss or asthenia. The musculoskeletal examination revealed painful and mobile subcutaneous right arm swelling with a soft consistency measuring 7 x 5 cm without overlying skin changes (Figure 1).

The pain was mild in intensity and exacerbated by physical exertion, with preserved shoulder and elbow range of motion. The neurovascular examination revealed no abnormalities. Lymph node examination was unremarkable.

**Figure 2:**
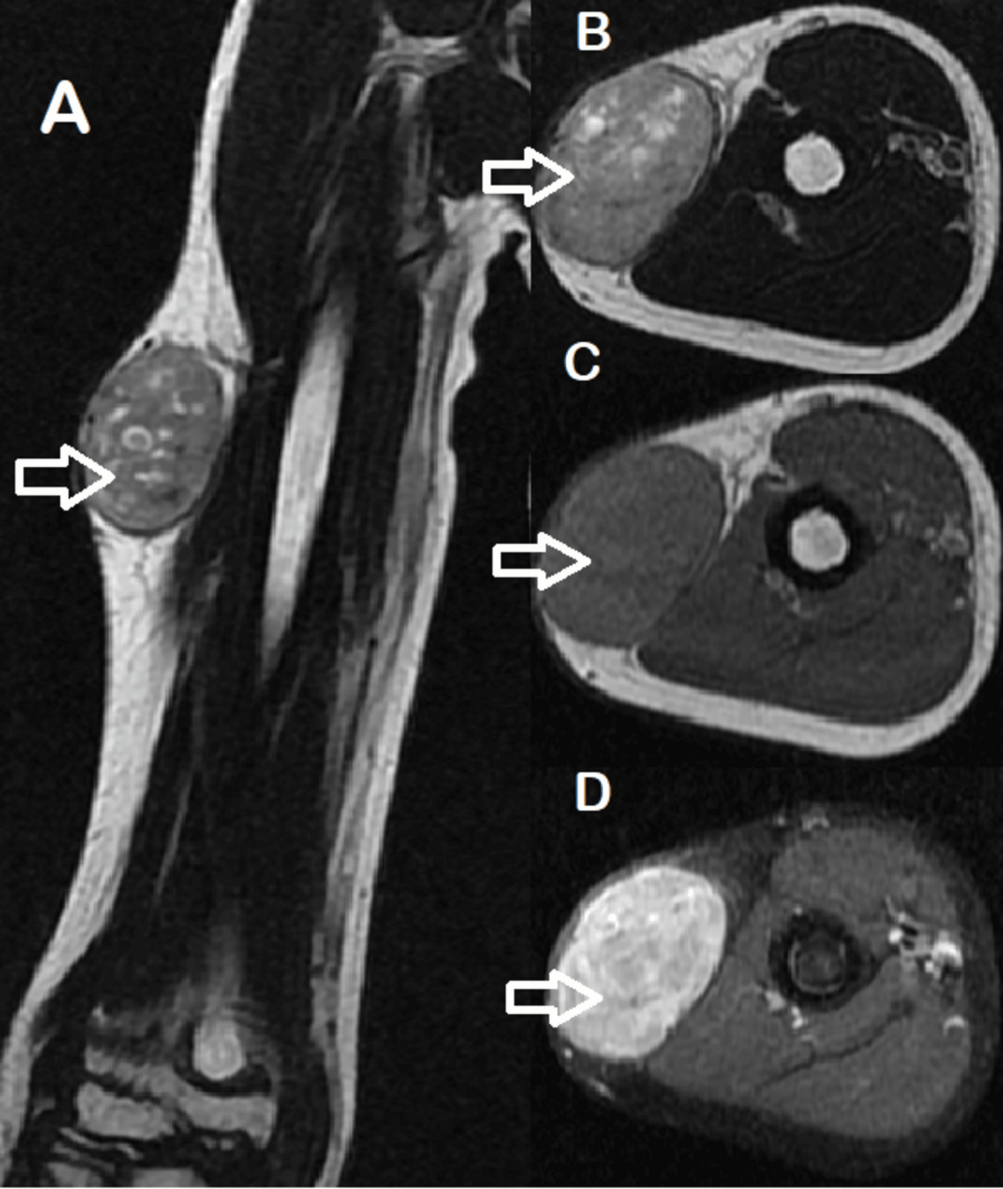
A, B: Coronal and transverse respectivly magnetic resonance imaging slice showing right arm subcutaneous tumoral swelling with regular seams on slight high T2 signal, measuring 67 x 47 mm (white arrow) with a low-signal-intensity capsule. C: Transverse magnetic resonance imaging showing right arm superficial mass exhinbiting heterogenous intermediate signal on T1 (white arrow) with a low-signal-intensity capsule. D: Transverse magnetic resonance imaging showing right arm lateral mass taking uniform contrast (white arrow ).

Magnetic resonance imaging (MRI) showed a well-circumscribed, oval mass in the right arm, measuring 67 × 47 mm with regular margins, exhibiting heterogeneous intermediate signal intensity on T2 and slight hyperintensity on T1 sequences. After contrast administration, the lesion demonstrated marked and relatively homogeneous enhancement. The mass was surrounded by a low-signal-intensity capsule on both T1- and T2-weighted sequences. It displaced the lateral head of the triceps brachii muscle without evidence of invasion and was located remote from the radial nerve (Figure 2).

**Figure 3:**
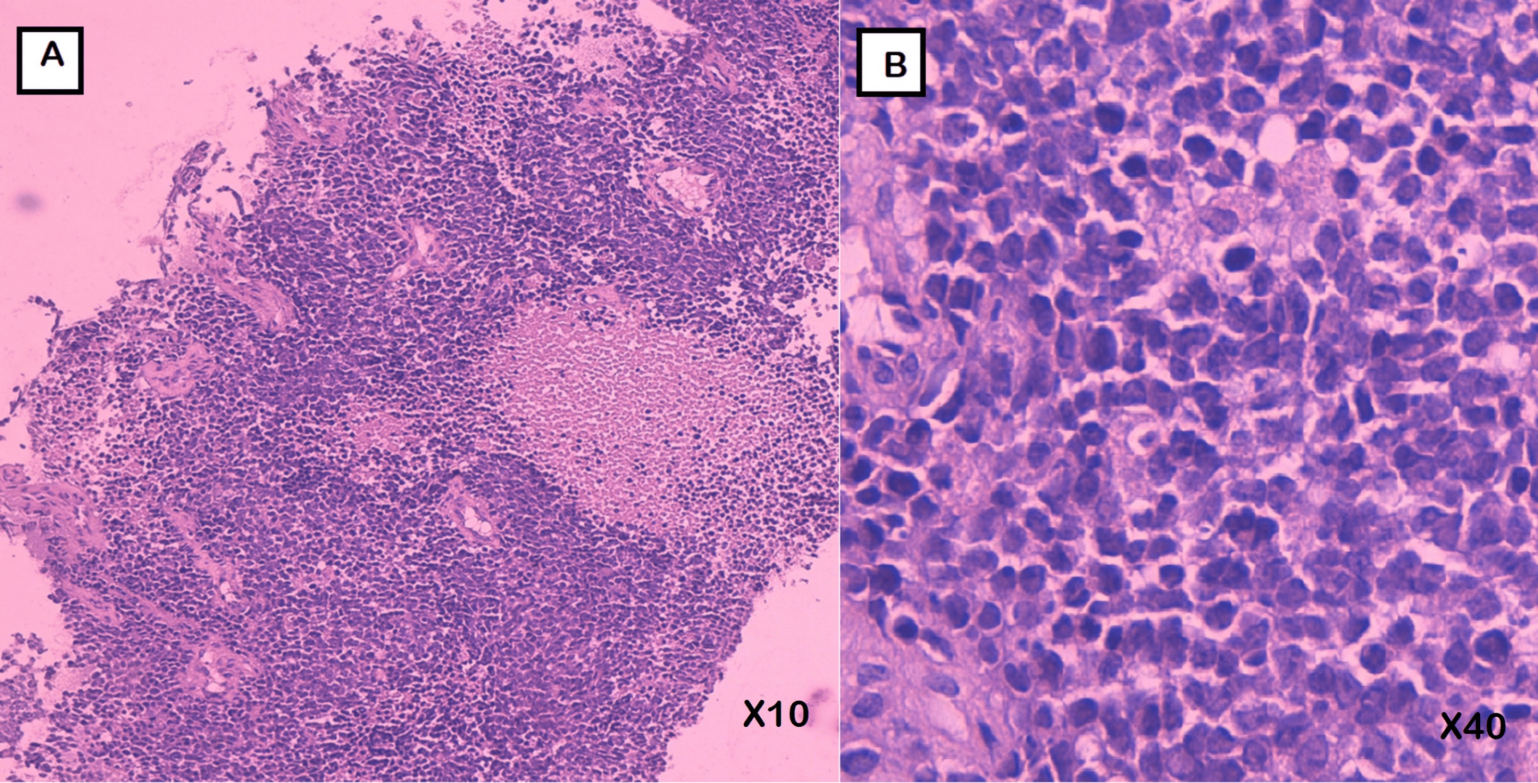
Tumoral proliferation displaying diffuse sheets of small round cells, with prominent vasculature and foci of necrosis (hematoxylin-eosin coloration, Magnification x10). B : High-power view showing atypical tumor cells , with a small and irregular nuclei, coarse chromatin, inconspicuous nucleoli, and moderate amount of eosinophilic cytoplasm (hematoxylin-eosin coloration, magnification x40).

In front of the characters of the MRI, the therapeutic decision was to carry out a surgical biopsy through a direct lateral approach after an extension assessment, objectifying the absence of metastasis. The anatomopathological examination found a blue, small, round cell tumor; an immunohistochemical complement by the marker CD99 was made, coming back positive. EWSR1 rearrangement was identified in 80% of tumor cell nuclei in fluorescence in situ hybridization, excluding other SRCTs such as small cell carcinoma, primitive neuroectodermal tumor, and embryonal rhabdomyosarcoma. The diagnosis of STES was retained (Figure 3).

**Figure 4:**
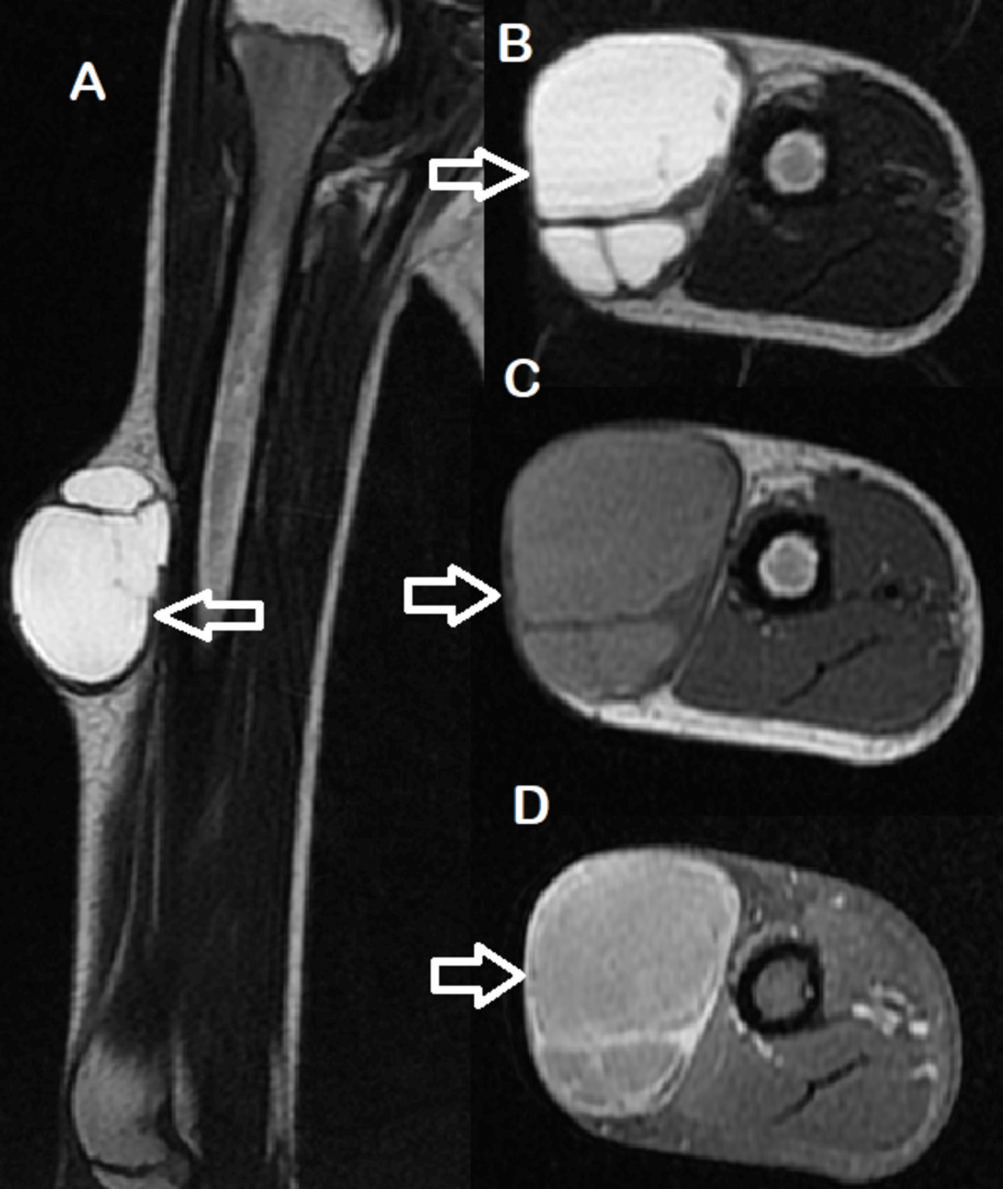
A, B: Coronal and transverse magnetic resonance imaging slices the lesion exhibiting cystic transformation, demonstrating a marked hyperintensity on T2 (white arrow) with internal septum and low-signal-intensity capsule. C: Transverse magnetic resonance imaging showing right arm superficial mass exhinbiting mild hyperintensity signal on T1 (white arrow) with a low-signal-intensity capsule. D: Transverse magnetic resonance imaging showing enhacement limited to the peripheral wall and septum and following contrast administration (white arrow).

The patient was initially treated by combined chemotherapy using the VDC/IE protocol: vincristine, doxorubicin, cyclophosphamide, ifosfamide, and etoposide. No adherence and tolerability incidents were observed. On follow-up MRI, the lesion exhibited cystic transformation, demonstrating marked T2 hyperintensity and mild T1 hyperintensity. A thick internal septum was observed, with enhancement limited to the peripheral wall following contrast administration (Figure 4).

In the second step, the patient underwent surgical resection of the lesion in accordance with oncological guidelines, removing the aponeurosis of the triceps brachii muscle and preserving the radial nerve. The postoperative course was uneventful. Based on the histological appearance, the diagnosis of STES was retained with clear excision margins and less than 20% residual viable tumor following chemotherapy.

Adjuvant chemotherapy was administered following the same protocol. After a two-year follow-up, the patient was asymptomatic and showed no signs of recurrence.

Table 1 shows the timeline summarizing the patient’s disease progression, treatment, and follow-up.

**Table 1:** A timeline summarizing the patient’s disease progression, treatment, and follow-up.

Six months before hospitalization	Hospitalization	Post-surgery care	After two years of follow-up
Right arm painful mass	Apyrexia and good general condition	Adjuvant chemotherapy	N evidence of recurrence or residual tumor
	Right arm painful swelling		
	First MRI		
	Biopsy		
	Histological diagnosis		
	Neoadjuvant chemotherapy		
	Evaluation MRI		
	Surgical resection		

## Discussion

ES is a rare aggressive tumor belonging to the family of primitive neuroectodermal tumours. It is the second most common bone cancer in terms of frequency after osteosarcoma. First described by Ewing James in 1921, it occurs mainly in young adults and children [1,7]. While ES usually originates in the bone, it can also arise in soft tissues. The most affected areas are the head, trunk, neck, and upper and lower limbs [8]. EES is a rare malignancy, occurring at an estimated rate of 0.1-0.4 cases per million people [9,10]. EES is uncommon before the age of 10 and after the age of 30. It is most frequently diagnosed during the second decade of life and shows no gender predominance; however, a female predominance is observed among elderly patients [2,5]. This case report presents a 14-year-old patient with no notable medical history, who developed a progressively enlarging right arm mass that was diagnosed as EES.

EES lacks distinctive clinical manifestations; the most common presenting symptoms are a rapidly enlarging mass and pain occurring in approximately 90% of patients. About two-thirds present with a palpable mass, and 20% present with fever, which may be misdiagnosed as infectious conditions, such as abscess or osteomyelitis [3,11]. In our case, it was a very limited mass and painful, and the fever was absent, which allowed us to eliminate an infectious pathology.

Imaging findings in EES are nonspecific and depend on tumor location and imaging modality. On plain radiographs, EES may appear as a soft-tissue mass with varying degrees of calcification [3]. Both MRI and CT are essential for characterizing the lesion, assessing local extension, and evaluating for metastases. On CT, EES typically presents as a well-defined soft-tissue mass with heterogeneous density, which may reflect calcification, necrosis, or hemorrhage [12,13]. MRI provides superior soft-tissue analysis, with EES demonstrating variable T1 signal intensity and T2 hyperintensity. The lesion frequently enhances following contrast administration [14]; however, assessment may occasionally be confounded by surrounding edema or muscle denervation, potentially leading to overestimation of tumor boundaries [15].

The biopsy is a crucial step to make the EES diagnosis; however, it must meet the following conditions: best performed by the surgeon who will achieve the surgical resection, avoid damage to vascular structures to reduce the risk of distant desimination, and the surgical approach must be performed in such a way to allow the excision of the whole path during the surgical resection of the mass, and if it not possible the incision must be approached and marked to be included in future radiation [16].

The diagnosis of EES is confirmed histologically, typicall findings associated with EES are small, round, blue cells with clear or eosinophilic cytoplasm and round nuclei [16, 17]. On immunohistochemistry, EES shows strong expression of CD99. However, this marker lacks specificity and cannot exclude other differential diagnoses, such as small cell osteosarcoma, lymphoma, rhabdomyosarcoma, or mesenchymal chondrosarcoma. Detection of an EWS gene translocation by fluorescence in situ hybridization (FISH) constitutes the defining feature of ES. The t(11;22)(q24;q12) translocation, found in about 90% of cases, generates the EWS-FLI1 fusion gene, leading to the production of a chimeric protein [16,18]. In our case, the EWSR1 rearrangement was identified in 80% of tumor cell nuclei in fluorescence in situ hybridization.

Given the rarity of STES, no specific recommendation exists for the treatment [19]. Management relies on multimodal therapy. Neoadjuvant chemotherapy reduces tumor size, facilitates complete surgical excision, and treats potential micrometastatic disease. Womer RB et al highlighted the benefit of chemotherapy according to the protocol VDC/IE in a randomized controlled trial for localized forms of ES [20]. Wide surgical excision with negative margins is crucial, particularly when preservation of vital structures is required. Adjuvant chemotherapy and radiotherapy in selected cases reduce recurrence risk. Prognosis depends on tumor size, location, presence of metastases, and response to therapy [19]. In our patient, the lesion was localized, margins were clear, and follow-up imaging at 24 months demonstrated no recurrence, highlighting the importance of early diagnosis, aggressive multimodal therapy, and careful surgical planning.

## Conclusions

ES is a common bone tumor seen during the first two decades of life. However, the soft tissue as an ES primary site is exceedingly rare and must be considered when evaluating a slowly enlarging soft tissue mass, especially in the limbs. STES management is multidisciplinary, with chemotherapy as a first-line treatment to reduce tumor size and make the surgical resection easier.
